# A Mobile Ecological Momentary Assessment Tool (devilSPARC) for Nutrition and Physical Activity Behaviors in College Students: A Validation Study

**DOI:** 10.2196/jmir.5969

**Published:** 2016-07-27

**Authors:** Meg Bruening, Irene van Woerden, Michael Todd, Stephanie Brennhofer, Melissa N Laska, Genevieve Dunton

**Affiliations:** ^1^Arizona State UniversityPhoenix, AZUnited States; ^2^University of MinnesotaMinneapolis, MNUnited States; ^3^University of Southern CaliforniaLos Angeles, CAUnited States

**Keywords:** validation study, ecological momentary assessment, nutritional status, physical activity, sedentary activity, emerging adults

## Abstract

**Background:**

The majority of nutrition and physical activity assessments methods commonly used in scientific research are subject to recall and social desirability biases, which result in over- or under-reporting of behaviors. Real-time mobile-based ecological momentary assessments (mEMAs) may result in decreased measurement biases and minimize participant burden.

**Objective:**

The aim was to examine the validity of a mEMA methodology to assess dietary and physical activity levels compared to 24-hour dietary recalls and accelerometers.

**Methods:**

This study was a pilot test of the SPARC (Social impact of Physical Activity and nutRition in College) study, which aimed to determine the mechanism by which friendship networks impact weight-related behaviors among young people. An mEMA app, devilSPARC, was developed to assess weight-related behaviors in real time. A diverse sample of 109 freshmen and community mentors attending a large southwestern university downloaded the devilSPARC mEMA app onto their personal mobile phones. Participants were prompted randomly eight times per day over the course of 4 days to complete mEMAs. During the same 4-day period, participants completed up to three 24-hour dietary recalls and/or 4 days of accelerometry. Self-reported mEMA responses were compared to 24-hour dietary recalls and accelerometry measures using comparison statistics, such as match rate, sensitivity and specificity, and mixed model odds ratios, adjusted for within-person correlation among repeated measurements.

**Results:**

At the day level, total dietary intake data reported through the mEMA app reflected eating choices also captured by the 24-hour recall. Entrées had the lowest match rate, and fruits and vegetables had the highest match rate. Widening the window of aggregation of 24-hour dietary recall data on either side of the mEMA response resulted in increased specificity and decreased sensitivity. For physical activity behaviors, levels of activity reported through mEMA differed for sedentary versus non-sedentary activity at the day level as measured by accelerometers.

**Conclusions:**

The devilSPARC mEMA app is valid for assessing eating behaviors and the presence of sedentary activity at the day level. This mEMA may be useful in studies examining real-time weight-related behaviors.

## Introduction

The majority of nutrition and physical activity (PA) assessments are subject to recall and social desirability biases, which can result in over- or under-reporting of behaviors [1,2]. For example, studies of dietary intake in adolescents and young adults have shown that people generally overestimate or underestimate their own consumption [3]. When self-reporting PA behaviors, young people tend to overestimate the time spent in, and the intensity of, PA efforts [4,5]. As such, many nutrition and PA measures suffer from low validity [6], resulting in limited interpretability of findings. There is a need to understand the nutrition and PA behaviors of young people through reliable and valid measurement tools that do not impose a high level of burden on participants or high costs to researchers.

Ecological momentary assessments (EMAs) limit measurement biases associated with self-reported recall data. As described by Shiffman et al [7] and Stone et al [8], EMAs involve sampling strategies that assess phenomena in the moment they occur in the participant’s natural environment, and they have at least three major advantages over traditional measurement tools for diet and PA: (1) avoidance of recall bias by collecting data in real time or near real time; (2) maximizing ecological validity by assessing behaviors in the environments where they occur; and (3) fine-grained temporal resolution, enabling analysis of behavior as it unfolds over time. A review of studies comparing EMA with traditional long-term recall-based methods points to EMA being better able to generate more valid results when researchers are interested in understanding a person’s experience as it occurs rather than their retrospective impressions of the experience [7]. In the context of eating and PA behavior assessment, the use of EMA could also lead to decreased participant burden, potentially yielding higher rates of compliance and lower rates of missing data.

Emerging technologies and changes in how people use technologies have created opportunities to assess behaviors as the behaviors occur. With increasing use and ownership of mobile phones, mobile technologies are valuable assets in behavioral health research [2,9,10]. Several studies have shown the usefulness and effectiveness of mobile technology-based EMAs (mEMAs), including mobile phone apps, texting, and personal digital assistants (PDAs) [10-15]; mEMAs are almost exclusively used in EMA research these days (as opposed to paper-and-pen and desktop computer EMAs), particularly in research with young people [15].

College students are an understudied population in regards to weight and weight-related behaviors and management [16]. The National Institutes of Health (NIH) has encouraged technology-driven weight management interventions for young adults due to the deficit of efforts for this critical population in transition [17,18]. mEMAs, particularly those using mobile phones, may be particularly useful for studying the behavior of today’s young adults, as these youth tend to make frequent and extensive use of mobile phones, owing in part to being the first generation to grow up completely with mobile technologies [19]. Because of their development stage, stressors, and ever-changing priorities, college students can be a difficult population to study over time [20], and finding ways to maximize compliance is critical. The use of mEMA in young adult research is limited, especially when focusing on nutrition and PA. Studies using mEMA methods in young people have tended to focus on substance use and other harmful behaviors (eg, tobacco use, marijuana use, binge eating) [11]. A few studies have validated mEMA for PA assessment in elementary and adolescent age groups [2,12]; however, to our knowledge, no study has validated mEMA for PA assessment in older adolescent/young adult (aged 16-21 years) populations or for dietary assessment in any population. In this study, we sought to examine the validity of a mEMA app, *devilSPARC*, in assessing dietary and PA behaviors with a college student sample.

## Methods

### Study Design

The SPARC (Social impact of Physical Activity and nutRition in College) study was a large-scale NIH-funded study that aimed to determine the mechanisms by which friendship networks and interpersonal connections impact weight and weight-related outcomes. In the formative phase of the study, participants answered EMA prompts asking about their current nutrition and PA behaviors using the mEMA app, devilSPARC, as well as validated measures of diet [20] and/or accelerometry across a 4-day period during the 2014-2015 academic year. Participants provided written consent prior to enrollment and were offered incentives of up to US $80 for their completion of the pilot study. All study protocols were approved by Arizona State University’s Institutional Review Board.

### Participants

College freshmen and assigned community mentors (resident assistants) at Arizona State University from two residence halls were recruited for participation. Inclusion criteria were (1) enrollment at Arizona State University and (2) living in target residence halls. For those participants who were interested in participating but did not own an Android or iOS mobile phone, a Motorola Moto G was loaned to them for use during the duration of the study. The resulting sample was 109 participants: 68 participants who provided mEMA and 24-hour dietary recalls only, 17 students who provided mEMA PA reports and accelerometry assessments only, and 24 participants who completed both protocols (92 dietary recalls, 41 accelerometry assessments).

### Measures

#### The devilSPARC Mobile Ecological Momentary Assessment App

The mEMA software was designed specifically for this study and implemented on Android- or iOS-compatible mobile phones. For each 4-day period of data collection (Wednesday-Saturday), participants received a total of 32 short message service (SMS) text message prompts to complete mEMA surveys via the devilSPARC app. Each day, participants received seven “real-time” prompts per day (n=28 total) and one retrospective prompt per day (n=4). Real-time prompts asked participants what they were doing in the moment before they received the prompt and retrospective prompts asked participants to recall what they did in the past 3 hours. A random, interval-contingent schedule was used for the mEMA prompts. Twice during each of the four established time periods per day (9 am-12 pm, 12 pm-3 pm, 3 pm-7 pm, and 7 pm-10 pm) the system prompted participants to complete a brief survey. In order to ensure the momentary nature of the mEMA, participants were allotted 35 minutes to respond to the prompt by completing a 1-minute survey, with the survey being available for 5 minutes prior to, and 30 minutes after, the text message prompt. Outside of these times, the mEMA surveys were not available to complete on the app. On average, the latency time (time between the sending of the SMS prompt to the completion of the survey on the mEMA app) was 7.25 minutes for participants completing the dietary validation and 6.90 minutes for participants completing the PA validation. Trained research assistants downloaded the devilSPARC mEMA app to each participant’s mobile phone and provided demonstrations on how to use the devilSPARC app.

All SMS text prompts were sent directly to participants’ mobile phones using Web service application programming interfaces (APIs) provided by Twilio, a cloud communications company. Through Twilio’s API, a series of six local long codes (10-digit phone numbers) were used to send text messages to participants. Each long code was randomly assigned to a participant based on his or her participant identification number. Any failed text messages were sent two more times, for a total of three attempts. The text message included motivational text with an embedded link that would open a survey on the app. Data were transferred instantaneously to the study host server every time the user’s phone contacted the central server (eg, on submission of a survey or opening of the home screen).

**Figure 1:**
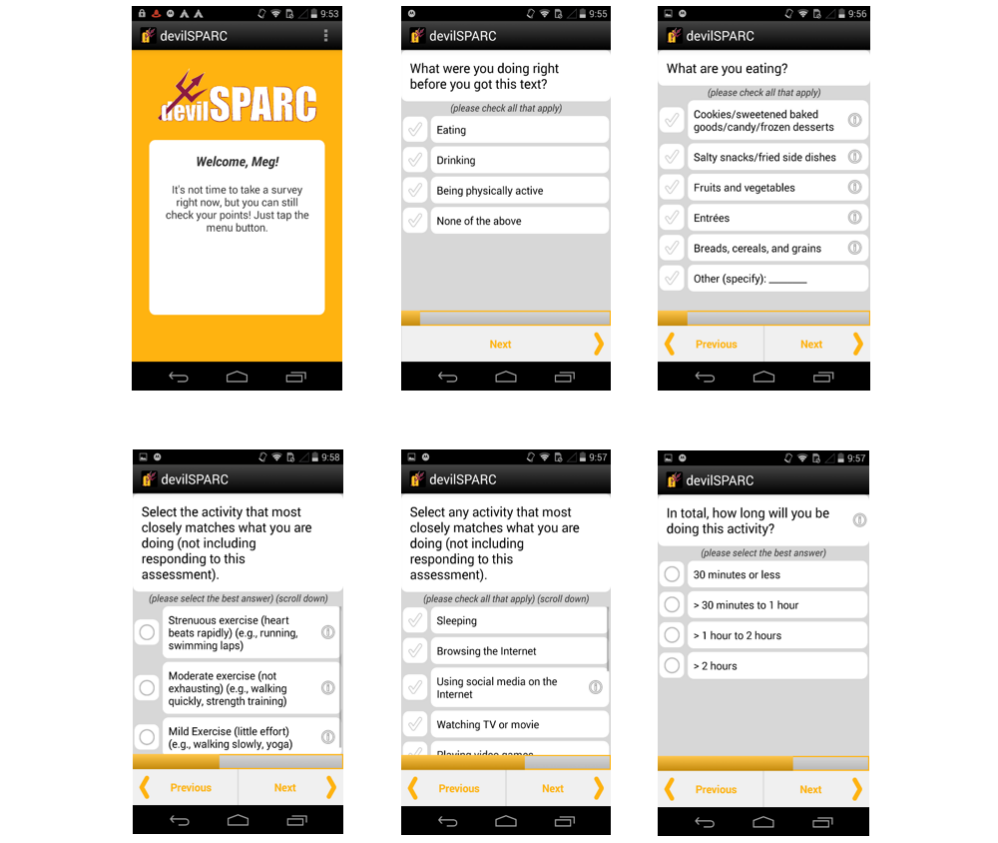
Screenshots of the eating and physical activity behaviors assessed in the devilSPARC mEMA app.

Figure 1 includes screenshots of the real-time mEMA items, which included the assessment of eating, drinking, PA and sedentary behaviors, and activities. The sequence of items measured varied based on a participant’s response to the first question, “What were you doing right before you got this text?” Participants could select all of the following that applied: eating, drinking, being physically active, or none of the above. If participants selected eating, then they were asked to identify food groups they were eating: (1) cookies/sweetened baked goods/candy/frozen desserts (sweets); (2) salty snacks/fried side dishes (salty foods); (3) fruits and vegetables; (4) entrées, (5) breads, cereals, and grains (breads/grains); and (6) other. These food groups were identified in previous research as types of food frequently consumed by college students [21]. If participants selected PA, then an adapted version of the Godin-Shepard measure of self-reported PA [22] was shown, and participants were instructed, “Select the activity that most closely matches what you are doing: strenuous exercise (heart beats rapidly), moderate exercise (not exhausting), and mild exercise (little effort).” If a participant did not select that they were being physically active, then it was assumed that they were involved in sedentary activity. Participants were then asked to respond to the following item: “Select any activity that most closely matches what you are doing (not including responding to this assessment).” Response options included sleeping, browsing the Internet, using social media, watching TV or a movie, playing video games, texting/snapchatting, attending class/doing homework/studying/reading, working, hanging out, and other (specify).

#### Dietary Recall

The online version of the Automated Self-Administered 24-hour (ASA24) dietary recall system, a validated measure of self-reported dietary intake, was used to assess participants’ food and beverage intake over the previous 24 hours [23]. The ASA24 utilizes the US Department of Agriculture’s (USDA) Automated Multiple Pass Method and measures intake by using the USDA’s Food and Nutrient Database for Dietary Studies. Participants were asked to complete 3 days of dietary recall (two weekdays and one weekend day). If participants reported at least one full day of biologically plausible data (ie, daily caloric intake between 500-5000 kilocalories [24-27]), their data were used in the analytic sample. Each food item reported in the ASA24 was coded to match the food groups in the mEMA: (1) sweets, (2) salty snacks/fried side dishes, (3) fruits and vegetables, (4) entrées (eg, pizza, sandwiches, lasagnas, chicken), (5) breads/grains, and (6) other.

#### Accelerometry

Actigraph, Inc (model GT3X+) accelerometer devices provided an objective measure for participants’ PA. A 60-second epoch for summing counts and the Freedson et al [28] cut points were used to classify PA levels (sedentary <100 counts per minute [CPM], light 100-1951 CPM, moderate 1952-5724 CPM, and vigorous ≥5725 CPM) for 30 minutes prior to and after the mEMA prompt.

### Data Analysis

Analyses were specific to dietary and PA data. Given the varied distribution for each of the food choices across both the mEMA and ASA24, comparisons between the mEMA and ASA24 responses at the moment of the mEMA response were not possible. However, because accelerometers capture PA every 60 seconds, direct comparisons between the PA level reported in the mEMA and the PA recorded by the accelerometer at the moment of the mEMA response were possible. The specific analyses are described subsequently.

#### Validating the Mobile Ecological Momentary Assessment Dietary Data

Participants’ data were excluded (n=15) from the analysis for days without (1) mEMA data with at least one food entry and (2) a biologically plausible ASA24 dietary recall. This resulted in an analytical sample of 92 from the 107 participants with ASA information (86.0%). The percentage match between the ASA24 and mEMA at both the daily level, and for time windows around the mEMA (ranging from 6 minutes to 8 hours, in 6-minute increments, on either side of the beginning of each mEMA report) were determined for each food type (sweets; salty snacks/fried side dishes; fruits and vegetables; entrées; and breads, cereals, and grains).

In day-level analyses, for each food type, the denominator for the match rate included the number of times the food type was reported via mEMA during the day; the numerator was the smaller of (1) the number of times the food type was reported in that day’s mEMA reports and (2) the corresponding ASA24 count for each participant. For example, if fruits and vegetables were reported four times via mEMA on a given day and three times in that day’s ASA24, this was recorded as three matches from a potential four (75% match rate). Conversely, if on a given day fruits and vegetables were reported three times via mEMA and four times in the corresponding ASA24, this would be recorded as three matches out of a potential three (100% match rate). For the time window analysis, the denominator for the match rate represented every mEMA response with the food type recorded; the numerator was the number of times the food type recorded in the mEMA was also in the ASA24 within the given time window.

Chi-square tests were used to determine if match rates differed between males and females, white and nonwhite participants, and participants with and without a Pell grant. Sensitivity and specificity values were computed for the time windows to determine the impact of increased windows size on the match rates. For the sake of conciseness, positive and negative likelihood ratios are not reported for each food type. Mixed effects logistic regression models with random participant-level intercepts were used to determine how well the endorsement of a food type in mEMA reports could be predicted from the endorsement of the same food type in the ASA24.

#### Validating Mobile Ecological Momentary Assessment Physical Activity Data

Participants’ mEMA responses were excluded (n=8) from the analysis if the accelerometer activity CPM values were zero for the 30 minutes before or after the mEMA response, or if the accelerometer had not been worn for at least 5 hours for the day of the mEMA. This resulted in an analytic sample of 41 from the 49 participants with accelerometer information (84%). Because accelerometer readings showed high minute-to-minute variability, the average accelerometer activity (CPM) value for the 5 minutes prior to the EMA response was used as the measure of accelerometer-derived activity. There were six parameters used to characterize agreement between the PA level as determined by accelerometer activity counts (sedentary, light, moderate, or vigorous, as described in the Measures section) and the PA level reported in the mEMA (sedentary, light, moderate, or strenuous). These parameters were (1) odds ratio: the odds that a participant’s accelerometer-derived activity level and mEMA-reported PA level were both at a specific PA level, compared to the odds a participant’s accelerometer-derived activity level was at the specified level, but the mEMA-reported PA level was not; (2) match rates: the percentage of times the accelerometer-derived activity level and reported mEMA level were the same for each mEMA level; (3) sensitivity (true positive rate): the percentage of times both the mEMA-reported PA levels and the accelerometer-derived activity level were at the same PA level for each accelerometer-derived PA level; (4) specificity (true negative rate): the percentage of times the mEMA-reported PA levels and the accelerometer-derived activity level were not at the specific PA level for each accelerometer-derived PA level; (5) positive likelihood ratio: the increase in the likelihood that a particular accelerometer-measured activity level (eg, moderate) was achieved, given that the same PA level was reported via mEMA; and (6) negative likelihood ratio: the decrease in the likelihood that a particular accelerometer-measured PA level (eg, moderate) was achieved, given that a *different* PA level was reported via mEMA. To determine if the distribution of the accelerometer-derived activity levels differed systematically with respect to mEMA-reported PA level, two-sample Kolmogorov-Smirnov tests were run. This nonparametric test compares the maximum vertical distance between the cumulative distribution functions of two distributions, with the *P* value corresponding to the probability that the distributions are the same (ie, small *P* values indicate greater discrepancy between the forms of the distributions). Mixed effects linear regression models with random participant-level intercepts were used to determine how well mEMA-reported PA categories predicted log transformed accelerometer-derived activity levels. All analyses were conducted using R statistical software version 3.2.3 (R Foundation for Statistical Computing, Vienna, Austria). Statistical significance was determined at *P*<.05.

## Results

Data from 92 participants were used in analyses examining validity of mEMA-reported dietary behavior (age: mean 18.83, SD 0.61 years; female: 67/92, 67%), and data from 41 participants were used in analyses aimed at examining the validity of mEMA-reported PA (age: mean 18.72, SD 0.50 years; female: 30/41, 73%; see Table 1).

### Dietary Validation

A total of 272 mEMA prompts and 607 ASA24 eating instances from 163 participant days were analyzed, including those from 17 participants who provided three days of ASA24 recall data, 37 participants who provided two days of data, and 38 participants who provided one day of data. Entrée was the most common food type reported in the mEMA (121/272, 44.5%), but was the least-reported food type reported in the ASA24 (294/607, 48.4%; see Table 2). At the day level, the percentage of occasions when a food type reported in the mEMA was also reported in the ASA24 ranged from 79% (95/121 entrées reported in the mEMA matched to ASA) to 94% (64/68 fruit and vegetables reported in the mEMA matched to ASA).

**Table 1:** Participant demographics in mEMA diet validation and PA validation.

Demographic variable		Diet validation (n=92)	PA validation (n=41)
**Gender, n (%)**			
	Male	30 (33)	11 (27)
	Female	62 (67)	30 (73)
Age (years), mean (SD)		18.83 (0.61)	18.72 (0.50)
**Race/ethnicity, n (%)**			
	White only	54 (59)	21 (51)
	Black only	4 (4)	4 (10)
	Mixed/other	17 (18)	5 (12)
	Hispanic	17 (18)	11 (27)
Pell grant status (yes), n (%)		29 (32)	18 (44)
**Major, n (%)**			
	Humanities	14 (15)	8 (20)
	Natural sciences	44 (48)	18 (44)
	Social sciences	22 (24)	9 (22)
	Other	12 (13)	6 (15)
**Year in college, n (%)**			
	First	86 (93)	41 (100)
	Second	2 (2)	0 (0)
	Third	4 (4)	0 (0)

**Table 2:** Number and percentage of times each food type was observed at the daily level for the mEMA and ASA24, and match rate at the daily level for each food type.

Self-reported food group	mEMA, n (%) (n=272)	ASA24, n (%) (n=607)	Match rate (%)
Bread/grains	55 (20)	392 (65)	89
Entrée	121 (44)	294 (48)	79
Fruit and vegetables	68 (25)	347 (57)	94
Salty foods	54 (20)	426 (70)	80
Sweets	45 (17)	404 (67)	91

**Figure 2:**
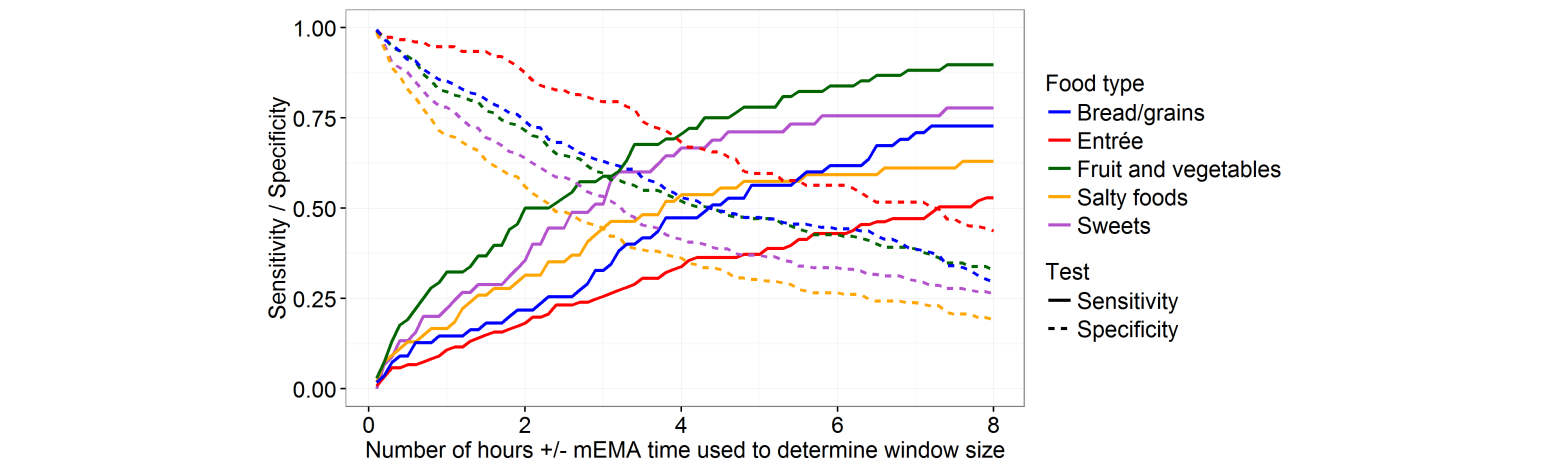
Sensitivity and specificity for each food type with increasing time window size.

Increased times on either side of the mEMA response resulted in increased specificity and decreased sensitivity (see Figure 2). Although participants reported the same foods in the mEMA and the ASA24, they were not accurate with the time they reported foods in the ASA24. In general, entrées had the lowest match rate across all the time windows, and fruits and vegetables had the highest match rate. There was no significant difference between the mEMA and ASA24 match rates by gender, race/ethnicity, or Pell grant status (data not shown). No significant associations between mEMA-reported food types and ASA24 reports were observed in the mixed model results (data not shown).

### Physical Activity Validation

A total of 694 mEMA surveys with valid accelerometer values across the 41 participants were included in the analysis. Table 3 presents the frequency and match rate of the mEMA activity and corresponding accelerometer-derived activity. Sedentary or light PA were the most often mEMA-reported activity levels (628/694, 90.5% of mEMA reports). Approximately 95% (656/694) of the accelerometer-derived activity levels corresponded with sedentary or light PA. Of the 26 mEMA responses reporting vigorous PA, only one participant’s accelerometer-derived activity levels indicated vigorous intensity.

**Table 3:** Cross-tabulation of frequencies of mEMA-reported and accelerometer-derived physical activity levels in 41 participants (n=694 mEMA reports).

mEMA-reported activity level	Accelerometer-derived activity level, n				Total of mEMA reports, n (%)
	Sedentary	Light	Moderate	Vigorous	
Sedentary	340	209	16	0	565 (81.4)
Light	19	37	7	0	63 (9.1)
Moderate	11	20	9	0	40 (5.8)
Vigorous	2	18	5	1	26 (3.8)
Total of accelerometer counts, n (%)	372 (53.6)	284 (40.9)	37 (5.3)	1 (0.1)	694 (100)

The odds of a participant having their accelerometer-derived activity level match their reported PA level were significant for mEMA-reported sedentary PA (OR 4.69, 95% CI 3.00-7.32), light PA (OR 2.27, 95% CI 1.32-3.88), and moderate PA (OR 6.30, 95% CI 2.65-14.95) Due to only one participant having vigorous accelerometer values, odds were not computed for vigorous activity. The match rates were highest for mEMA-reported sedentary and light PA (340/565, 60.3% and 37/63, 58.7%, respectively), and lowest for moderate PA (9/40, 22.5%) and vigorous PA (1/26, 3.8%).

We also conducted sensitivity and specificity between mEMA-reported activities and accelerometer-derived activities, for each respective PA intensity level. Specificity and positive likelihood ratio values were lower for mEMA-reported sedentary PA (specificity=30%, positive likelihood ratio=1.31) than mEMA-reported light (specificity=94%, positive likelihood ratio=2.05), moderate (specificity=95%, positive likelihood ratio=5.16), and vigorous (specificity=96%, positive likelihood ratio=27.72) PA. Sensitivity values were highest for mEMA-reported sedentary (91%) and vigorous PA (100%), and lowest for mEMA-reported light (13%) and moderate PA (24%); negative likelihood ratio values were lowest for mEMA-reported sedentary (0.29) and vigorous PA (0.00), and highest for mEMA-reported light (0.93) and moderate PA (0.79).

**Figure 3:**
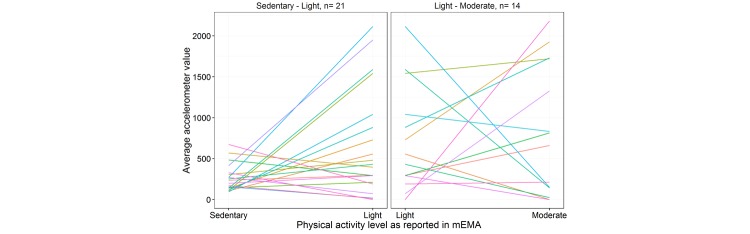
Within-person difference in accelerometer values by reported physical activity levels.

The difference in participants’ average accelerometer-derived activity levels was not consistent across the mEMA levels. For example, as illustrated in Figure 3, for eight of 21 participants who reported sedentary and light PA via mEMA, the average accelerometer-derived activity level was *lower* on occasions when they reported light PA than on occasions when they reported being sedentary. Similarly, for six of the 14 participants who reported both light and moderate PA, on occasions when moderate PA was reported average, accelerometer-derived activity levels were lower than occasions when light PA was reported.

To examine the differences in distributions of accelerometer-derived activity levels for different reported mEMA PA levels, we conducted Kolmogorov-Smirnov tests. Results indicated a significant difference in the distribution of the accelerometer-derived activity counts for mEMA-reported sedentary and nonsedentary PA levels (*P*<.001; Table 3). The difference between the distribution of the accelerometer-derived activity counts for mEMA-reported light and vigorous PA was also significant (*P*=.02). No difference between the distribution of the accelerometer-derived activity counts were seen between mEMA-reported light and moderate PA occasions, or moderate and strenuous PA occasions.

**Table 4:** Kolmogorov-Smirnov results examining whether the accelerometer-derived activity count distributions for each pair of mEMA levels could be from the same distribution.

Activity levels as reported in mEMA	Activity levels as reported in mEMA, *P*		
	Light (n=63)	Moderate (n=40)	Strenuous (n=26)
Sedentary (n=565)	<.001	<.001	<.001
Light (n=63)	—	.13	.02
Moderate (n=40)	—	—	.26

To more closely examine the association between mEMA-reported intensity of PA to accelerometer-measured levels, we estimated differences in distributions of logged accelerometer activity count values for pairs of mEMA-reported PA levels using mixed linear regression models (with repeated observations nested within participants) (Table 5). There was a significant (*P*<001) difference between logged activity count values for mEMA-reported sedentary and nonsedentary accelerometer occasions, but no significant difference in logged counts across mEMA-reported light, moderate, and strenuous PA occasions (*P*=.84, *P*=.05, and *P*=.10, respectively). For example, when comparing mEMA-reported sedentary versus light PA occasions, activity counts were higher for mEMA-reported light PA occasions than for sedentary occasions *(P<*.001). The estimated average increases in logged accelerometer activity counts between sedentary and nonsedentary occasions were 1.71, 1.81, and 2.79 for light, moderate, and vigorous PA, respectively, corresponding to differences of 178, 201, and 603 CPM, respectively, in raw count values.

**Table 5:** Estimated differences, 95% confidence intervals, and *P* values for pairwise comparisons of logged accelerometer activity counts between mEMA-reported PA levels.^a^

mEMA-reported PA level	mEMA-reported PA level					
	Light		Moderate		Vigorous	
	Diff (95% CI)	*P*	Diff (95% CI)	*P*	Diff (95% CI)	*P*
Sedentary	1.71 (1.09-2.33)	<.001	1.81 (1.04-2.57)	<.001	2.79 (1.85-3.73)	<.001
Light	–		0.10 (–0.85-1.04)	.84	1.08 (–0.01-2.18)	.05
Moderate	–		–		0.99 (–0.19-2.17)	.10

^a^ Estimates from mixed models adjusted for nonindependence of repeated within-person observations.

## Discussion

### How Well Does the devilSPARC App Measure Eating and Physical Activity Behaviors?

This study assessed the validity of the devilSPARC mEMA app as a tool for assessing eating and PA behaviors among young adults compared to online dietary recall and accelerometry methodologies. Diet and PA assessment methods commonly used in current research settings often require high levels of cost and personal effort for participants. Few objective assessments of dietary quality and intake are available; self-report remains the norm in observational studies. Objective assessments of PA tend to use expensive devices. The mEMA had high match rates with day-level reported dietary intake as measured by 24-hour recall. For PA behaviors, mEMA reports differentiated sedentary from nonsedentary activity, but these reports did not accurately distinguish among objectively measured PA levels. These findings suggest that the devilSPARC mEMA app had relatively high criterion validity with food choices and for distinguishing between sedentary versus nonsedentary activity.

Research has demonstrated that methods for dietary recall are subject to significant compliance, self-reporting, and recall errors [3,28]. These analyses excluded several participants because of biologically implausible values in the 24-hour dietary recall measure. With the exception of one, all exclusions were a result of participants reporting daily intakes of less than 500 kilocalories. Anecdotally, many participants reported frustration with the functionality of the ASA24 website and the amount of time it took to complete the recall. As with findings from other studies, we expect that the 24-hour recall data reported here underrepresents dietary intake and misestimates the time at which participants consumed food [29-32]. The potential lack of adherence to the 24-hour recall protocol may explain why the sensitivity for food choices increased over time, from approximately 70% for 8-hour windows surrounding the time at which a given food was reported on the mEMA to 10% for half-hour windows (ie, lower match in shorter window). Although still relatively high, the measure of entrées showed the lowest match rate at 79% between the mEMA and the ASA24; this is likely because of the lack of specificity of what participants perceived as an entrée. Our results demonstrated that with just a few questions, devilSPARC mEMA may be able to assess food choices with significantly less burden than the self-administered 24-hour dietary recall for each eating occasion, particularly given there was relatively high construct validity.

Reports of light, moderate, and vigorous levels of PA from the mEMA did not correspond to intensity of PA as measured objectively through accelerometry. The proportion of participants reporting an activity level that corresponded to the accelerometer decreased with increasing PA level. Social desirability and/or perception biases may be at play with these results. Social desirability is often related to over-reporting of activity duration and intensity [1,33,34]. Other research has also reported that the percent agreement in validating mEMA is highest for sedentary activity [35]. In addition, individuals who are heavier or are less fit may perceive an activity to be more intense due to increased respiration and heart rates [36]. Because established accelerometer activity cut points do not take into account body weight or current fitness level, energy expenditure may vary across individuals who are engaging in the same amount of objectively measured PA.

### Limitations

To our knowledge, this is the first study to validate a mEMA tool assessing eating and PA behaviors among young people; however, several limitations should be considered. The devilSPARC app did not assess quantity of foods or specific details of the foods (ie, brand or type), as this would have added to the response burden; the devilSPARC tool assessed broad behaviors and was therefore not able to yield information about total caloric intake, macronutrients, or micronutrients. Given the relatively equal distribution of food choices captured, we were able to assess a variety of commonly consumed foods, including healthy and unhealthy food choices, for young adults in college. The devilSPARC mEMA tool was designed to assess behaviors in the moment; as such, it could not represent total dietary intake or total PA. In addition, because the mEMA and the 24-hour recall relied on self-reports, participants’ reporting biases and idiosyncratic interpretations of mEMA questions could have increased measurement error. Despite verbal and written directions to wear the accelerometer at all times except when swimming and bathing, there is a possibility that participants removed the accelerometer when participating in vigorous activities, such as contact sports, which would have improved our match rate between the mEMA and more vigorous activities. Also, although the sample was relatively diverse in terms of race/ethnicity, almost all participants were college freshmen; thus, these findings may not be generalizable beyond young adult populations.

### Conclusions

This new mEMA tool is valid for assessing eating behaviors and the presence of PA. With very brief surveys spaced through the day, this mEMA tool may reduce participant burden as compared to 24-hour dietary recall or PA recall instruments. The mEMA builds on previous measures of assessing eating and PA, including a wide range of foods.

## Abbreviations

API: application programming interfaces
CPM: counts per minute
EMA: ecological momentary assessment
mEMA: mobile-based ecological momentary assessment
NIH: National Institutes of Health
PA: physical activity
SMS: short message service
SPARC: Social impact of Physical Activity and nutRition in College
USDA: US Department of Agriculture
